# Aggravated pulmonary injury after subarachnoid hemorrhage in PDGF-B^ret/ret^ mice

**DOI:** 10.1186/s41016-020-00193-2

**Published:** 2020-06-01

**Authors:** Pengyu Pan, Jie Qu, Qiang Li, Rongwei Li, Yang Yang, Shilun Zuo, Xin Liu, Hua Feng, Yujie Chen

**Affiliations:** 1 Department of Neurosurgery, Southwest Hospital, Third Military Medical University, Chongqing, 400038 China; 2 Department of Neurosurgery, General Hospital of Shenyang Military Command, Shenyang, 110016 China; 3 State Key Laboratory of Trauma, Burn and Combined Injury, Third Military Medical University, Chongqing, 400038 China; 4 Chongqing Key Laboratory of Precision Neuromedicine and Neuroregenaration, Southwest Hospital, Third Medical University, Chongqing, 400038 China

**Keywords:** Platelet-derived growth factor B, Pulmonary injury, Subarachnoid hemorrhage, Lung-specific surfactant protein

## Abstract

**Background:**

Recent advances in surgical and neuroprotective strategies could effectively manage the pathophysiological progression of subarachnoid hemorrhage (SAH). However, pulmonary dysfunction frequently occurs in SAH patients with an increased risk of unsatisfactory outcomes. Based on the similar microvascular structures in the blood-air barrier and blood-brain barrier and possible brain-lung crosstalks, we believe that pericytes may be involved in both neurological and pulmonary dysfunction after SAH.

**Methods:**

In our experiments, platelet-derived growth factor B (PDGF-B) retention motif knockout (PDGF-B^ret/ret^) mice and adeno-associated virus PDGF-B were employed to show the involvement of pericyte deficiency and PDGF-B expression. Neurological score, SAH grade, hematoxylin-eosin staining, and PaO2/FiO2 ratio analysis were performed to evaluate the neurological deficits and pulmonary functions in endovascular perforation SAH models at 24 h after surgery, as well as western blotting and immunofluorescence staining for underlying molecular expressions.

**Results:**

We found that neonatal PDGF-B^ret/ret^ mice exhibited pulmonary atelectasis 12 h after birth. Further investigation showed a decrease in PaO_2_/FiO_2_ and lung-specific surfactant proteins in adult PDGF-B^ret/ret^ mice. These dysfunctions were much worse than those in wild-type mice at 24 h after SAH. PDGF-B overexpression alleviated pulmonary dysfunction after SAH.

**Conclusions:**

These results suggested pulmonary dysfunction after SAH and the pivotal role of PDGF-B signaling for the pathophysiological process and future therapeutic targets of pulmonary injury treatment after SAH. Further studies are needed for pathophysiological investigations and translational studies on pulmonary injuries after SAH.

## Background

Despite years of effort, subarachnoid hemorrhage (SAH) is still a complicated intracranial hemorrhagical stroke subtype associated with high morbidity and fatality, resulting in substantial loss to society. Recent advances in surgical and neuroprotective strategies could effectively manage the pathophysiological progression of SAH. However, pulmonary dysfunction frequently occurs in SAH patients with an increased risk of unsatisfactory outcomes [1], which was reported in approximately 10% of SAH survivors and almost half of deceased patients [2] without a proper therapeutic strategy. Neurogenic pulmonary edema is reported to be an important pathophysiological process that induces pulmonary dysfunction [3]. Based on the similar microvascular structures between blood-brain barrier and blood-air barrier and possible brain-lung crosstalks, we believe that pericytes may be involved in both neurological and pulmonary dysfunction after SAH and platelet-derived growth factor B (PDGF-B)-related signaling may not only control pericyte recruitment in cerebral microvasculature [4] but also act as an independent neurogenic mechanism for pulmonary dysfunction. Previous studies demonstrated that PDGF-B and PDGF receptor beta (PDGFR-β) were associated with the development of normal postnatal lung [5], as well as the incidence of cerebral vasospasm complication after SAH. So, we knockout and bred the PDGF-B retention motif in C57BL/6 genetic background mice (PDGF-B^ret/ret^ mice) to investigate PDGF-B involvement in pulmonary injury after SAH.

## Methods

As previously reported [6], we employed male PDGF-B^ret/ret^ mice as knockout mice in our experiments, which were bred by the Shanghai Biomodel Organism Science and Technology Development Company Limited. Male Heterozygous PDGF-B^ret/+^ mice and wild-type PDGF-B^+/+^ mice were used as littermate controls. A total of 197 mice (4 weeks old, weighing 22 to 30 g) and 21 neonatals were employed in this study. All experimental procedures related to mice were approved by the Laboratory Animal Welfare and Ethics Committee of the Third Military Medical University (no. AMUWEC2019449), complied with the guidelines by the National Institutes of Health 2011 Guide for the Care and Use of Laboratory Animals, and were presented in the format of the Animals in Research: Reporting In Vivo Experiments (ARRIVE) guidelines for purpose of replacement, refinement, and reduction of animals in research. Mice were bred in specific pathogen-free rooms of the Animal Center of Southwest Hospital, with controlled temperature and humidity 1 week before experiments and were given free access to water and food, along with half-day light/dark cycles.

Hematoxylin-eosin (HE) staining and western blotting were performed in neonatals at P0.5. Neurological score, SAH grade, hematoxylin-eosin staining, and PaO_2_/FiO_2_ ratio analysis were performed to evaluate the neurological deficits and pulmonary functions in endovascular perforation SAH models at 24 h after surgery, as well as western blotting and immunofluorescence staining for underlying molecular expressions. Details of these methods and statistical analysis are described as follows.

### SAH model

As previously reported, endovascular perforation surgery was performed to induce SAH in mice [7]. All mice in the present study were intraperitoneally anesthetized with 1% sodium pentobarbital at the 40 mg/kg dosage. Briefly, start in the common carotid artery bifurcation, we slight migrated a sharpened 5–0 nylon suture into the distal internal carotid artery until slightly obstruct. After piercing the bifurcation between proximal middle cerebral artery and anterior cerebral artery, the resistance decreases. Then nylon suture was quickly extracted to restore blood flow and spread into intracranial subarachnoid space to mimic SAH onset. The mice in sham procedure had the same maneuver without perforating their vessels.

### SAH grade assessment

Success of mice SAH models were evaluated by the grading the subarachnoid blood volume as previously described [8]. Briefly, according to the blood clot in subarachnoid, the basilar cistern was partitioned into 6 segments with a score from 0 to 3 for each segment. When calculating the total scores, add up the scores of all areas (maximum SAH score = 18). Due to the limited subarachnoid blood clot and mild neurological deficits, SAH mice with a score < 8 should be excluded from this study.

### Adeno-associated virus administration

For in vivo adeno-associated virus (AAV) administration, the coding regions of *pdgfb* (738 bp) from C57BL/6J mouse cDNA were cloned into the AAV ITR-containing plasmid CMV-EGFP-2A-MCS-3FLAG, which became CMV-EGFP-2A-*pdgfb*. Recombinant AAV9 containing CMV-EGFP-2A-MCS-3FLAG (AAV9-EGFP) and CMV-EGFP-2A-*pdgfb* (AAV9-*pdgfb*) was manufactured in triple-transfection, helper-free procedures, and then purified by OBIO Technology (Shanghai) Corp., Ltd. A total of 75 μL sterile PBS solution of 10^12^ v.g. AAV9-*pdgfb* was administered by intratracheal intubation to each mouse 4 weeks before SAH to overexpress full-length PDGF-B protein in the lung, and AAV9-EGFP served as a control.

### Hematoxylin and eosin staining

Hematoxylin and eosin staining were performed as described previously [9]. Lung specimens were removed and post-fixed for a minimum of 24 h in 4% paraformaldehyde solution, then paraffin embedded and slice into the 8-μm-thick sections on a vibratome machine. These sliced sections were infiltrated in xylene for deparaffinizing and then in decreasing gradient ethanol solutions for rehydration. Then, the sliced sections were placed in glass slides and stained with the hematoxylin dye and eosin dye for further observation.

### PaO_2_/FiO_2_ ratio analysis

To evaluate pulmonary function after SAH, the PaO_2_/FiO_2_ (P/F) ratio was calculated as below. At 24 h after mimic SAH onset, mice were similarly intraperitoneally anesthetized with 1% sodium pentobarbital at the dosage of 40 mg/kg and endotracheally intubated with a 22-gauge catheter for mechanical ventilation with 21% oxygen. The respiratory frequency was 18 breaths per minute. Arterial blood sample was collected from the carotid artery and measured by following the operator’s manual in blood gas analyzer.

### Western blot

Western blot analysis for protein expressions were performed as reported previously in our laboratory [7]. Lung specimens were collected after mice sacrifice and lysed in 20 mM Tris for following standard western blot analysis. Primary antibodies were list as follow: anti-SP-B antibody (Abcam, Cambridge, MA), anti-SP-C antibody (Abcam, Cambridge, MA). Primary α-tubulin antibody from Beyotime Biotechnology (Shanghai, China) was loaded as the internal control for normalization between different groups. The corresponding secondary antibodies were then incubated and imaged with an ECL+Chemiluminescent Kit (Amersham Bioscience, Arlington Heights, IL) to identify the immune bands. Further analysis was performed by densitometry quantified with Quantity One software (Bio-Rad, Berkeley, CA).

### Immunofluorescence staining

The protein expression in lung specimens was also evaluated by immunofluorescence staining on fixed frozen sections similar to HE staining as previously described [7]. Brain sliced sections on coverslips were fixed in 4% paraformaldehyde. Samples were blocked by 5% donkey serum prior to incubation with primary antibody overnight with the following antibodies: anti–SP-C (Abcam, Cambridge, MA) at 4 °C overnight, followed by fluorescein-conjugated antibodies for immunofluorescence (Beyotime Biotechnology, Shanghai, China). Then, the sliced sections were placed on glass slides for laser-confocal microscope observation.

### Statistical analysis

SPSS 18 (SPSS China, Shanghai, China) was used for the statistical analyze in the present study. Data are presented in the format of mean ± standard deviation (mean ± SD). Neurobehavioral scores were analyzed by using chi-square tests. One-way analysis of variance (ANOVA) plus Tukey’s multiple comparisons test was used for multiple comparisons of more than three groups. If the *P* < 0.05, the comparison among groups was considered significantly different.

## Results

### PDGF-B involvement in pulmonary injury in neonatal mice

**Fig. 1:**
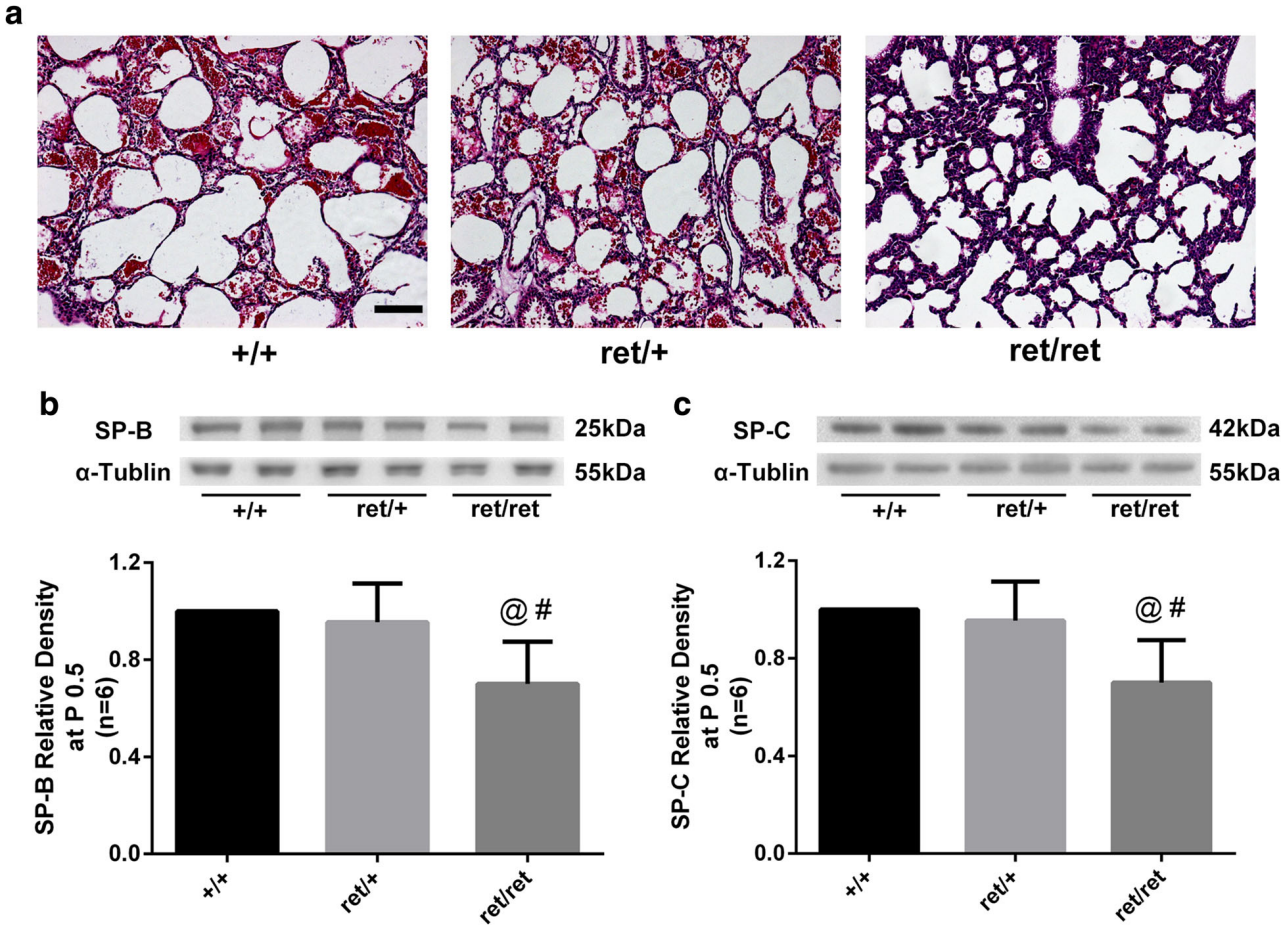
Pulmonary injury in neonatal PDGF^ret/ret^ mice. **a** Representative HE staining images of the lung parenchyma in neonatal PDGF^ret/ret^ mice and controls at 12 h after birth. Representative bands and quantitative graphs of **b** SP-B and **c** SP-C expressions in pulmonary specimens at 12 h after birth. Relative densities were controlled with α-tubulin. Those western blot bands were collected under the same conditions and cropped form better exhibition effect. *N* = 6, @: vs. +/+ *P* < 0.05, #: vs. ret/+ *P* < 0.05. Scale bar 100 μm\

Hematoxylin-eosin staining indicated that neonatal PDGF-B^ret/ret^ mice exhibited pulmonary atelectasis at 12 h after birth (P0.5), which was less severe in PDGF^ret/+^ mice (Fig. 1a). This phenomenon was associated with significantly lower SP-B and SP-C expressions in the pulmonary tissues of neonatal PDGF-B^ret/ret^ mice at P0.5 (*P* < 0.05, Fig. 1b, c).

**Fig. 2:**
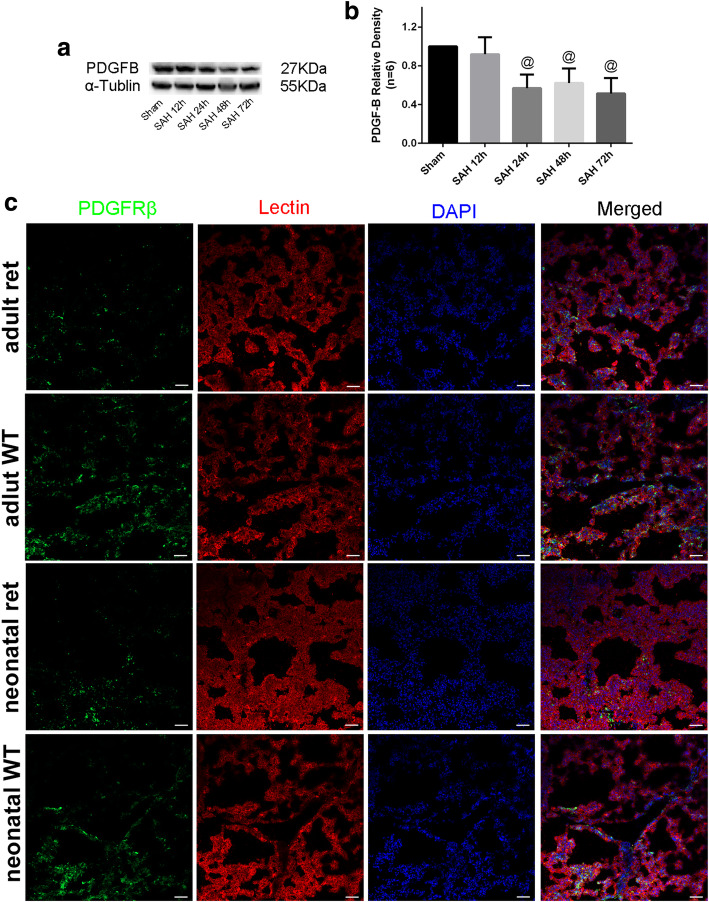
PDGF-B expression in the lungs after SAH and pericytes in wild-type and PDGF^ret/ret^ mice at both the neonatal and adult stages. **a** Immunoblot bands and **b** quantitative graphs of PDGF-B expression in lung specimens at 24 h after mimic SAH onset. Relative densities were controlled with α-tubulin. Those immunoblot bands were collected under the same conditions and cropped form better exhibition effect. **c** Representative immunofluorescence images of PDGFRβ (green) and Lectin (red) expressions in the lung parenchyma of wild-type PDGF^+/+^ and PDGF^ret/ret^ mice at both the neonatal and adult stages. Relative densities were controlled with α-tubulin. *N* = 6, @: vs. sham *P* < 0.05. WT: wild-type, ret: PDGF^ret/ret^, Scale bar 50 μm

In addition, PDGF-B protein expression was significantly decreased at 24 to 72 h after mimic SAH in adult PDGF-B^+/+^ mice (*P* < 0.05, Fig. 2a, b). Immunofluorescence indicated that the pericytes in the lungs were greatly reduced in PDGF-B^ret/ret^ mice at both the neonatal and adult stages compared to those in PDGF-B^+/+^ mice (Fig. 2c). These images also indicated that the pericyte deficits in the lung tissues of PDGF-B^ret/ret^ neonatal mice were not normalized during adulthood (Fig. 2c).

### Pulmonary dysfunction after SAH in adult PDGF-B^ret/ret^ mice

**Fig. 3:**
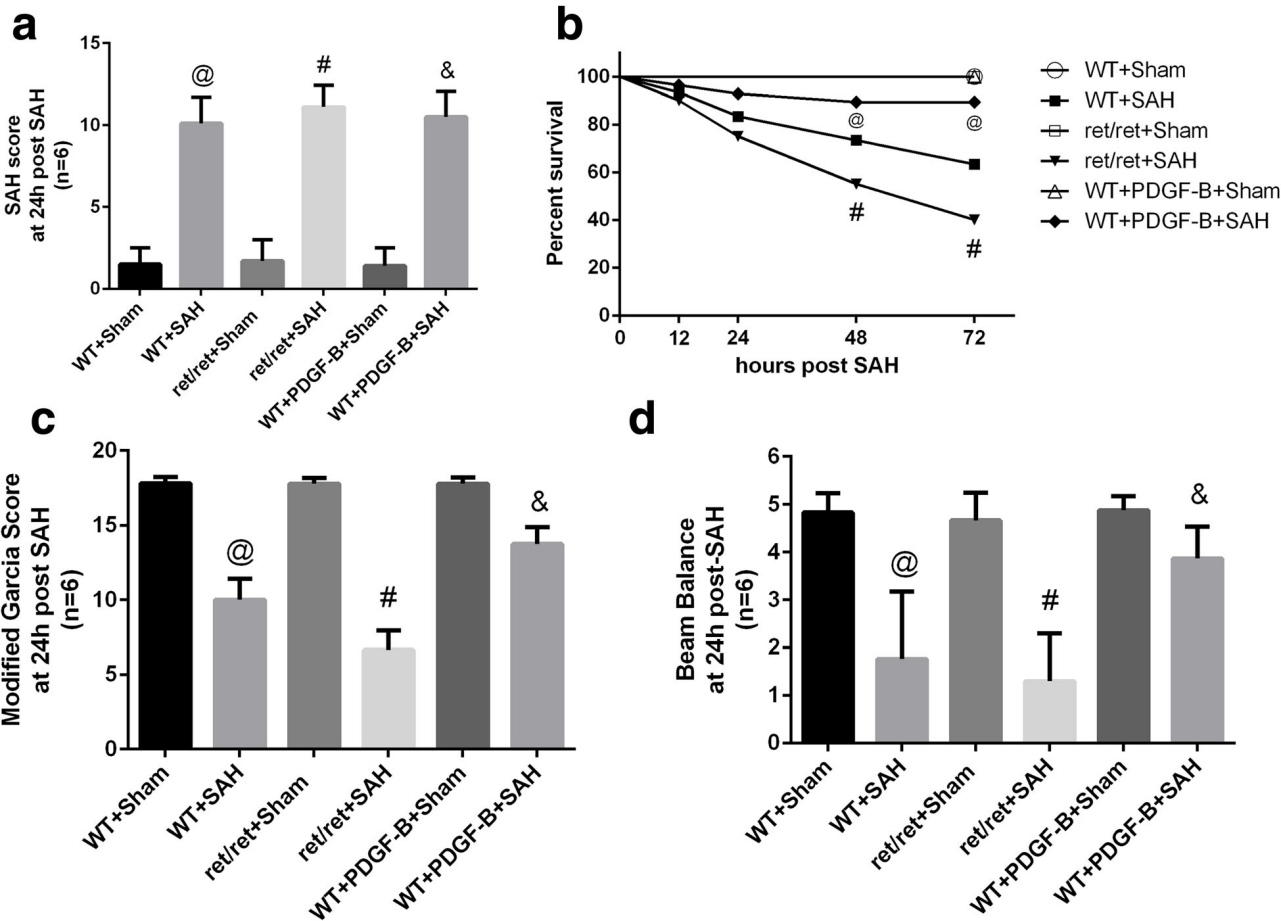
Survival percent and neurological functions after SAH. **a** The SAH grading of mice among groups at 24 h after SAH. **b** The survival percent at 12, 24, 48, and 72 h in each group. **c**, **d** Neurological evaluation scores of mice among groups at the timepoint of 24 h after mimic SAH onset. WT: wild-type, ret/ret: PDGF^ret/ret^, PDGF-B: AAV9-*pdgfb*. *N* = 20 for percent survival analysis, *N* = 6 for others, @: vs. WT + sham *P* < 0.05, & in panel **a**: vs. WT + PDGF-B + sham *P* < 0.05, & in other panels: vs. WT + SAH *P* < 0.05, # in panel **a**: vs. ret/ret + sham *P* < 0.05, # in other panels: vs. WT + SAH *P* < 0.05

**Fig. 4:**
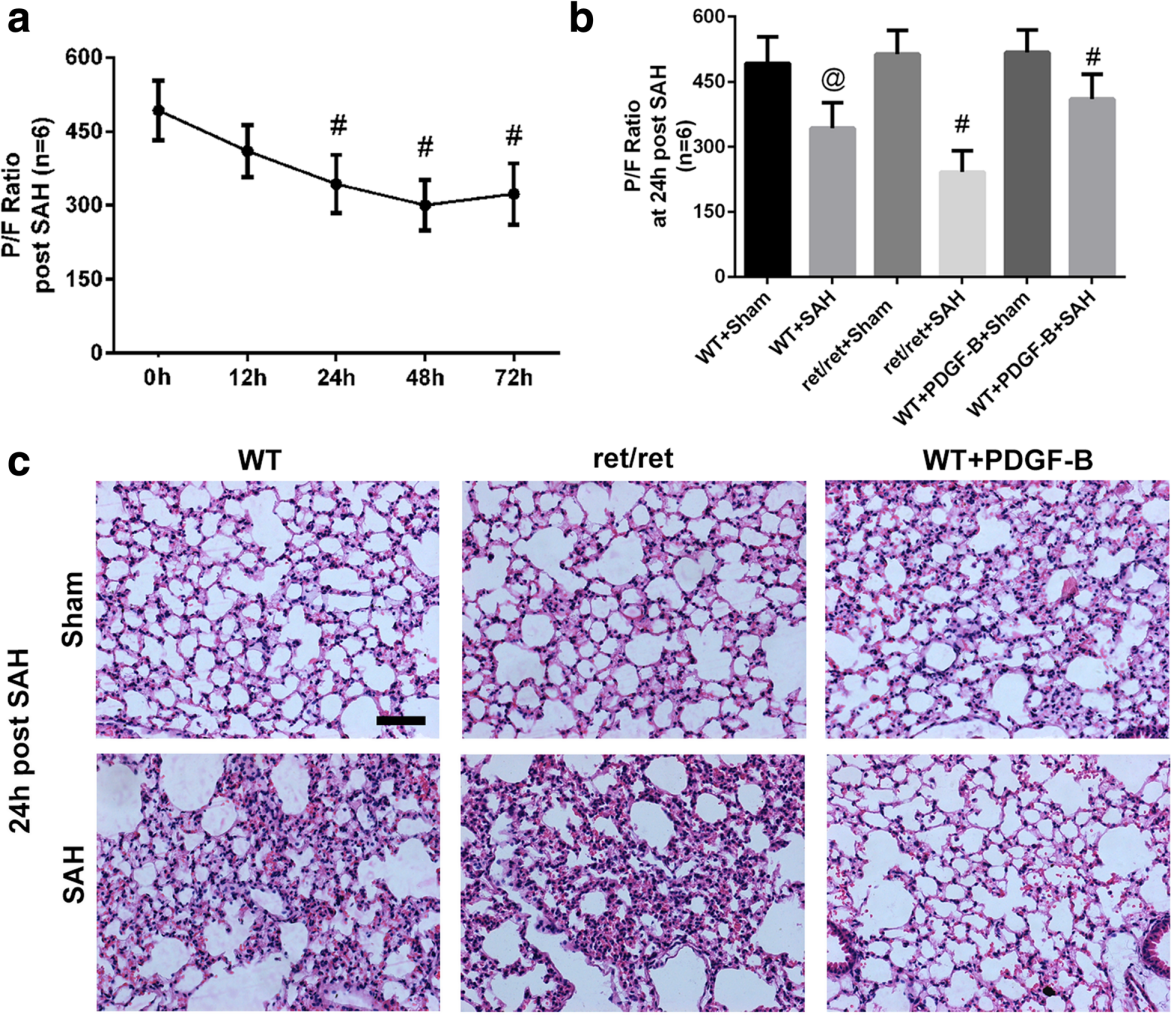
Pulmonary dysfunction after SAH in adult PDGF^ret/ret^ mice. **a** The time course of the PaO_2_/FiO_2_ ratio after SAH in wild-type mice. **b** The PaO_2_/FiO_2_ ratio at the timepoint of 24 h after mimic SAH onset. **c** HE staining pictures of the lung parenchyma. WT: wild-type, ret/ret: PDGF^ret/ret^, PDGF-B: AAV9-*pdgfb*, Scale bar 200 μm. *N* = 6, @: vs. WT + sham *P* < 0.05, # in panel **b**: vs. WT + sham *P* < 0.05, # in panel **a**: vs. sham *P* < 0.05

At adult stage, there is no mice died after sham-operated, and 13 mice died after SAH modeling, including 4 PDGF-B^+/+^ and 9 PDGF-B^ret/ret^ mice. And the grading scores have no significant differences to confirm the consistence SAH model induced in our study (*P* > 0.05, Fig. 3a). The survival rates of PDGF-B^ret/ret^ mice were much lower comparing to the PDGF-B^+/+^ mice at 48 or 72 h timepoints after mimic SAH onset (*P* < 0.05, Fig. 3b). Neurological deficits did not differ between PDGF-B^ret/ret^ and PDGF-B^+/+^mice at the timepoint of 24 h after mimic SAH onset (*P* > 0.05, Fig. 3c, d). PaO_2_/FiO_2_, a key indicator of pulmonary function, in the wild-type group was reduced at 24, 48, and 72 h after SAH versus the sham-operated mice (*P* < 0.05, Fig. 4a), which demonstrated pulmonary injury existing post-SAH. Further investigation demonstrated that PaO_2_/FiO_2_ in PDGF-B^ret/ret^ mice at 24 h after SAH was significantly lower comparing to the PaO_2_/FiO_2_ in PDGF-B^+/+^ mice (*P* < 0.05, Fig. 4b). These results indicated aggravated pulmonary injury after SAH in PDGF-B^ret/ret^ mice.

### Pulmonary dysfunction after SAH associated with low lung-specific surfactant proteins

**Fig. 5:**
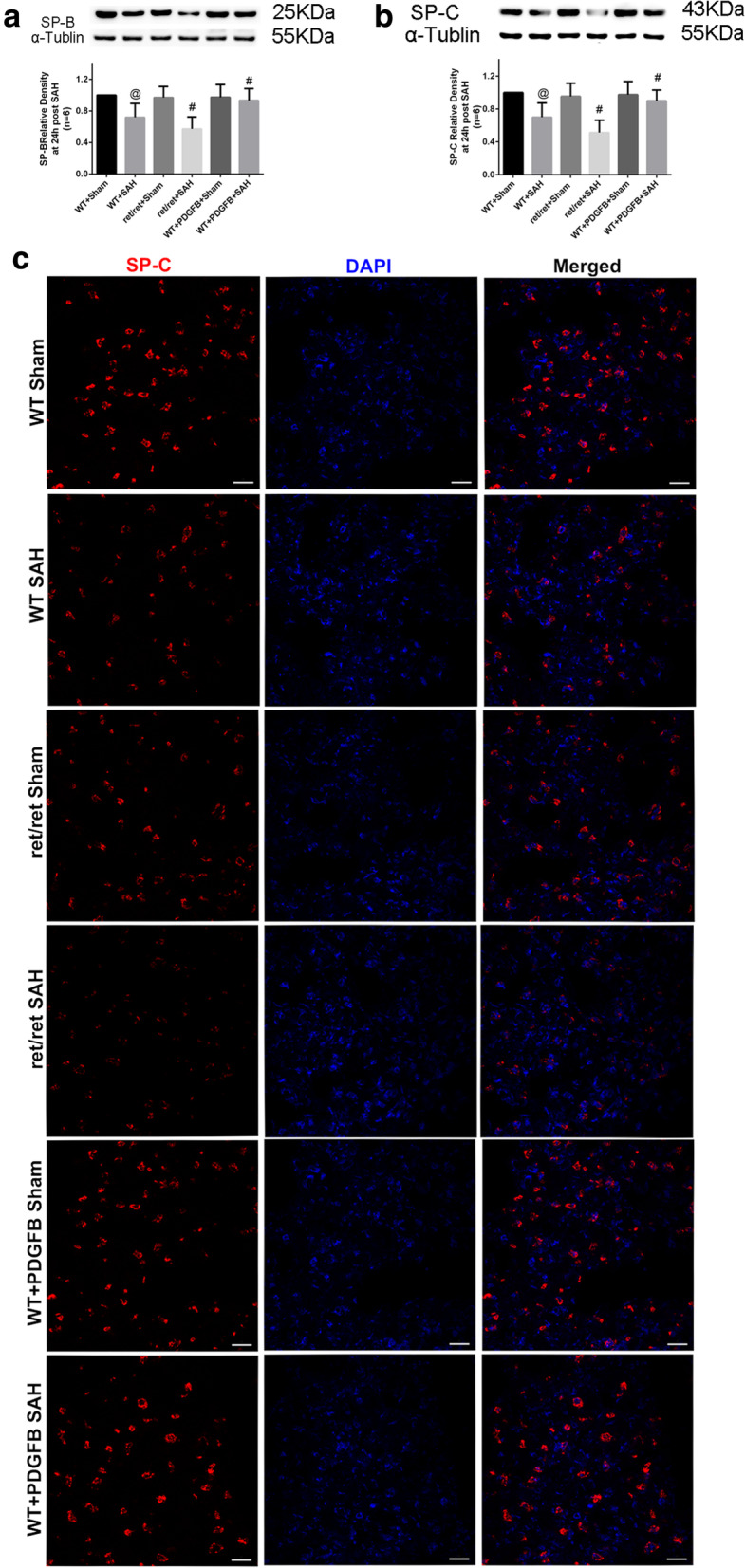
Expression of lung-specific surfactant proteins after SAH in adult PDGF^ret/ret^ mice. Immunoblot bands and quantitative graphs of **a** SP-B and **b** SP-C expressions in pulmonary specimens at 24 h after mimic SAH onset. Relative densities were controlled with α-tubulin. Those immunoblot bands were collected under the same conditions and cropped form better exhibition effect. **c** Representative immunofluorescence images of SP-C expression in lung parenchyma at 24 h after mimic SAH onset. WT: wild-type, ret/ret: PDGF^ret/ret^, PDGF-B: AAV9-*pdgfb*, *N* = 6. @: vs. WT + sham *P* < 0.05, #: vs. WT + sham *P* < 0.05. Scale bar 10 μm

Pulmonary autopsy showed similar pulmonary atelectasis at 24 h after SAH in PDGFB^ret/ret^ mice, meanwhile, the PDGFB^+/+^ SAH mice were much better at the same time point (Fig. 4c). Western blotting showed that much lower SP-B and SP-C expressions in pulmonary tissue after SAH, and much lower expressions in PDGF-B^ret/ret^ mice comparing with PDGF-B^+/+^ mice (*P* < 0.05, Fig. 5a, b). These results were confirmed with immunofluorescence staining, which showed less fluorescence intensity in PDGF-B^ret/ret^ mice comparing with PDGF-B^+/+^ mice at 24 h after SAH (Fig. 5c).

### Pulmonary dysfunction after SAH was alleviated after PDGF-B overexpression

To be more translational, we employed adeno-associated virus to overexpress PDGF-B in adult wild-type mice. The results indicated that PaO2/FiO2 was significantly increased in PDGF-B-overexpressing mice at 24 h after SAH compared to normal PDGF-B^+/+^ SAH mice (*P* < 0.05, Fig. 4b), which demonstrated that pulmonary injury after SAH was alleviated by PDGF-B overexpression. This phenomenon was also connected with increased SP-B and SP-C expressions in pulmonary tissue (*P* < 0.05, Fig. 5a–c). Meanwhile, as previously reported to be neuroprotective [10], PDGF-B overexpression significantly improved the survival percent and neurological outcomes after SAH in adult wild-type PDGF-B^+/+^ mice (*P* < 0.05, Fig. 3b–d).

## Discussion

In the present study, neonatal PDGF-B^ret/ret^ mice exhibited pulmonary atelectasis at P0.5, which confirmed the involvement of PDGF-B in pulmonary injury. Further investigation showed a decrease in PaO_2_/FiO_2_ and lung-specific surfactant proteins and evidence of pulmonary dysfunction after SAH in adult PDGF-B^ret/ret^ mice, which were much worse than wild-type mice at 24 h after SAH. PDGF-B overexpression alleviated pulmonary dysfunction after SAH. These results suggested pulmonary dysfunction after SAH and indicated the pivotal role of PDGF-B signaling for pathophysiological process and future therapeutic targets of pulmonary injury in SAH patients.

PDGFs serves as dimers of A, B, C, D single chains and ligands interact with its tyrosine-kinase alpha and beta receptors, short named as PDGFR-α and PDGFR-β. A previous study suggested that PDGF was induced in cerebrospinal fluid and vasculature, associated with an enhanced contractile response and vasospasm after SAH[11, 12]. PDGFA^−/−^ mice developed secondary lung emphysema after alveolar septation failure, which may be the result of low elastin expression [13]. Overexpress PDGF-A exhibited fatal pulmonary atelectasis at embryonic day 18.5 and could not survive after birth, with increased macrophages/eosinophils in airspaces and reduced elastin expression and emphysema [14]. Our results demonstrated that PDGF-B signaling was involved in pulmonary injury, with physiological changes in pulmonary atelectasis and reduced lung-specific surfactant protein. Consistently, PDGF-B antisense would inhibit fetal lung cell growth [15], suggesting a possible protective role of PDGF-B signaling for the lung development and repair. After exposure to a high concentration (60%) of oxygen, PDGF-B mRNA, but not PDGF-A, was increased in the lung parenchyma compared to the air-exposed lung after birth [5], indicating that PDGF-B might be more sensitive to hypoxia in the brain and lung after SAH. However, the fact that PDGF-B^ret/ret^ mice have lung defects at baseline from birth may somehow influence the evaluation of pulmonary dysfunction after SAH.

In our experiments, we knocked out retention motif of PDGF-B, which is the binding site of PDGF-B with the extracellular matrix [16]. Previous studies demonstrated that PDGF-B^ret/ret^ mice suffered cerebral vascular dysfunction by pericyte recruitment deficiency and subsequent breach on the blood-brain barrier after SAH [4, 17–20]. Similarly, the pericytes were covered with 26–40% microvessels in mature lungs [21], which might also be regulated by PDGF-B in the pathophysiological progression after SAH to cause pulmonary edema, previously considered to be neurogenic [22]. Based on our results, the PDGF-B signal played an important role in pulmonary injury after SAH, including pulmonary elastin expression, lung-specific surfactant proteins, and blood oxygen exchange. However, due to the limitations of the present study, we cannot exclude other possible mechanisms of pulmonary injury after SAH, such as FOXF1 maintaining endothelial barriers and preventing neurogenic edema after lung injury [23].

## Conclusion

PDGF-B might serve as a potential therapeutic target for pulmonary dysfunction after SAH and greatly change the situation of merely supporting strategies for this severe peripheral complication in SAH patients. Further studies are still needed for pathophysiological investigations and translational studies on pulmonary injuries after SAH.

## Data Availability

All data generated or analyzed during this study are included in this published article.

## Abbreviations

SAH: Subarachnoid hemorrhage
PDGF-A/B: Platelet-derived growth factor A/B
PDGFR-α/β: Platelet-derived growth factor receptor alpha/beta
PDGF-B^ret/ret^: PDGF-B retention motif knockout
ARRIVE: Animals in Research: Reporting In Vivo Experiments
AAV: Adeno-associated virus
P/F: PaO_2_/FiO_2_
SP-B/C: Lung-specific surfactant protein B/C

## References

[1] Veeravagu A, Chen YR, Ludwig C, Rincon F, Maltenfort M, Jallo J, Choudhri O, Steinberg GK, Ratliff JK (2014). Acute lung injury in patients with subarachnoid hemorrhage: a nationwide inpatient sample study. World Neurosurg.

[2] Lantigua H, Ortega-Gutierrez S, Schmidt JM, Lee K, Badjatia N, Agarwal S, Claassen J, Connolly ES, Mayer SA (2015). Subarachnoid hemorrhage: who dies, and why?. Crit Care.

[3] Vespa PM, Bleck TP (2004). Neurogenic pulmonary edema and other mechanisms of impaired oxygenation after aneurysmal subarachnoid hemorrhage. Neurocrit Care.

[4] Winkler EA, Bell RD, Zlokovic BV. Central nervous system pericytes in health and disease. Nat Neurosci. 2011;14:1398–405. 10.1038/nn.2946 PMC4020628 22030551

[5] Buch S, Han RN, Cabacungan J, Wang J, Yuan S, Belcastro R, Deimling J, Jankov R, Luo X, Lye SJ (2000). Changes in expression of platelet-derived growth factor and its receptors in the lungs of newborn rats exposed to air or 60% O(2). Pediatr Res.

[6] Lindblom P, Gerhardt H, Liebner S, Abramsson A, Enge M, Hellstrom M, Backstrom G, Fredriksson S, Landegren U, Nystrom HC (2003). Endothelial PDGF-B retention is required for proper investment of pericytes in the microvessel wall. Genes & Development.

[7] Chen Y, Zhang Y, Tang J, Liu F, Hu Q, Luo C, Tang J, Feng H, Zhang JH (2015). Norrin protected blood-brain barrier via frizzled-4/beta-catenin pathway after subarachnoid hemorrhage in rats. Stroke.

[8] Sugawara T, Ayer R, Jadhav V, Zhang JH (2008). A new grading system evaluating bleeding scale in filament perforation subarachnoid hemorrhage rat model. J Neurosci Methods.

[9] Li Q, Chen Y, Li B, Luo C, Zuo S, Liu X, Zhang JH, Ruan H, Feng H (2016). Hemoglobin induced NO/cGMP suppression deteriorate microcirculation via pericyte phenotype transformation after subarachnoid hemorrhage in rats. Sci Rep.

[10] Arimura K, Ago T, Kamouchi M, Nakamura K, Ishitsuka K, Kuroda J, Sugimori H, Ooboshi H, Sasaki T, Kitazono T (2012). PDGF receptor beta signaling in pericytes following ischemic brain injury. Curr Neurovasc Res.

[11] Shiba M, Suzuki H, Fujimoto M, Shimojo N, Imanaka-Yoshida K, Yoshida T, Kanamaru K, Matsushima S, Taki W (2013). Role of platelet-derived growth factor in cerebral vasospasm after subarachnoid hemorrhage in rats. Acta Neurochir Suppl.

[12] Borel CO, McKee A, Parra A, Haglund MM, Solan A, Prabhakar V, Sheng H, Warner DS, Niklason L (2003). Possible role for vascular cell proliferation in cerebral vasospasm after subarachnoid hemorrhage. Stroke.

[13] Bostrom H, Willetts K, Pekny M, Leveen P, Lindahl P, Hedstrand H, Pekna M, Hellstrom M, Gebre-Medhin S, Schalling M (1996). PDGF-A signaling is a critical event in lung alveolar myofibroblast development and alveogenesis. Cell.

[14] Li J, Hoyle GW (2001). Overexpression of PDGF-A in the lung epithelium of transgenic mice produces a lethal phenotype associated with hyperplasia of mesenchymal cells. Dev Biol.

[15] Liu M, Liu J, Buch S, Tanswell AK, Post M (1995). Antisense oligonucleotides for PDGF-B and its receptor inhibit mechanical strain-induced fetal lung cell growth. Am J Physiol.

[16] Abramsson A, Kurup S, Busse M, Yamada S, Lindblom P, Schallmeiner E, Stenzel D, Sauvaget D, Ledin J, Ringvall M (2007). Defective N-sulfation of heparan sulfate proteoglycans limits PDGF-BB binding and pericyte recruitment in vascular development. Genes Dev.

[17] Chen Y, Li Q, Tang J, Feng H, Zhang JH. The evolving roles of pericyte in early brain injury after subarachnoid hemorrhage. Brain Res. 1623;2015:110–22. 10.1016/j.brainres.2015.05.004 PMC4569518 25982598

[18] Moura DAP, Lemos RR, Oliveira JRM. New data from Pdfgb (ret/ret) mutant mice might lead to a paradoxical association between brain calcification, pericytes recruitment and BBB integrity. J Mol Neurosci. 2017;63:419–21. 10.1007/s12031-017-0992-z 29098547

[19] Villasenor R, Kuennecke B, Ozmen L, Ammann M, Kugler C, Gruninger F, Loetscher H, Freskgard PO, Collin L. Region-specific permeability of the blood-brain barrier upon pericyte loss. J Cereb Blood Flow Metab. 2017;37:3683–94. 10.1177/0271678X17697340 PMC5718326 28273726

[20] Abramsson A, Lindblom P, Betsholtz C. Endothelial and nonendothelial sources of PDGF-B regulate pericyte recruitment and influence vascular pattern formation in tumors. J Clin Invest. 2003;112:1142–51. 10.1172/JCI18549 PMC213487 14561699

[21] Sims DE, Westfall JA (1983). Analysis of relationships between pericytes and gas exchange capillaries in neonatal and mature bovine lungs. Microvasc Res.

[22] Saracen A, Kotwica Z, Wozniak-Kosek A, Kasprzak P (2016). Neurogenic Pulmonary Edema in Aneurysmal Subarachnoid Hemorrhage. Adv Exp Med Biol.

[23] Cai Y, Bolte C, Le T, Goda C, Xu Y, Kalin TV, Kalinichenko VV (2016). FOXF1 maintains endothelial barrier function and prevents edema after lung injury. Sci Signal.

